# A generalised framework for building complex networks of evolution equations

**DOI:** 10.1007/s00285-026-02346-w

**Published:** 2026-06-13

**Authors:** Irmand Leblond Mikiela Ndzoumbou, Valentina Lanza, M. A. Aziz-Alaoui, Arnaud Ducrot, Armel Andami Ovono

**Affiliations:** 1 Université Le Havre Normandie, Le Havre, France; 2 Department of Mathematics and Applications, 17009 ENS Libreville, Gabon

**Keywords:** Complex Network Modeling, Reaction-Diffusion Equations, Multi-Scale Diffusion, Predator-prey, Non-local coupling

## Abstract

We present a new approach to model partial differential equation (PDE) networks. The originality of our method lies in its ability to model various types of networks, including those whose nodes (domains) are non-identical, i.e. they can have various shapes and sizes. This approach makes it possible to model phenomena with possible connections both from within domains and from their boundaries, thus offering a general overview of transmission models. We propose a general model for the scalar case, demonstrating flow conservation in the network as well as the existence, uniqueness and positivity of the solution. In addition, we develop a model for the vector case, where we establish results similar to those of the scalar case, while adding an analysis on the global bounding of the solution in mass-conserving systems. We illustrate our approach with a concrete application to a predator-prey model in a network with two nodes, to which we apply all the obtained theoretical results. Finally, numerical simulations are provided to illustrate our theoretical findings.

**Supplementary Information:**

The online version contains supplementary material available at 10.1007/s00285-026-02346-w.

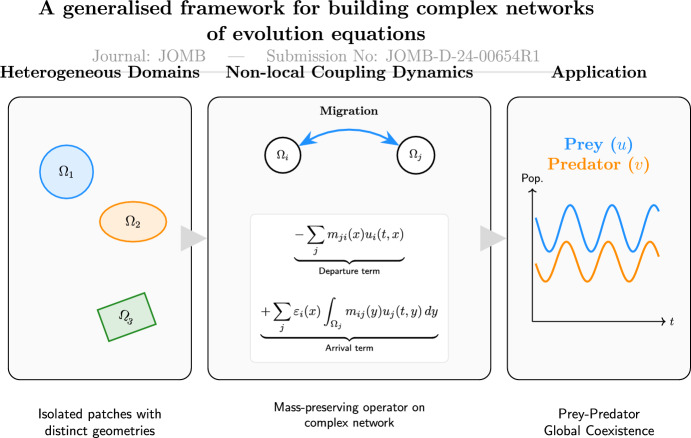

 

- A generalised framework for building complex networks of evolution equations
- Non-local terms model long-distance interactions between spatially disjoint domains
- Several properties of the network model can be derived from the ones of the model on the single node
- Diffusion, predation and migration of populations are effectively captured by these models

## Introduction

In this article, we consider a set of coupled PDEs defined on disjoint domains. These types of models can suitably describe the dynamics of invasive species, whether humans, animals, or plants. Movement between distinct domains (for example, between two islands) is modeled through mechanized transport mechanisms such as boats, airplanes, or trains. More generally, these models characterize phenomena taking place in different locations and interacting with each other. In particular, here we exploit the complex networks framework (Strogatz 2001), and each node of our network represents a distinct domain. On each domain a set of PDE describing our phenomenon (for instance, the spatio-temporal evolution of a population) is defined. A link between two nodes means that there is an interaction between the phenomena on the two corresponding domains. In the case of population migration, a network connection can represent a movement of individuals between the two domains.

In the literature, we often come across metapopulation models on multiple patches (Lieberthal et al. 2023; Xu et al. 2024; Arino 2009). The main objective of these ODE-type models is to describe the temporal evolution and the migration process between different domains. However, they provide no information on spatio-temporal dynamics within patches. David et al. (2020) proposed a hybrid PDE/ODE framework where the local dynamics of populations are linked through indirect interactions, in the context of airborne pathogen transmission. This study illustrates the importance of accounting for heterogeneous domains and partly inspired us. However, it remains focused on a particular case and does not provide a systematic methodology for constructing arbitrary PDE networks. In Besse and Faye (2021); Bertaglia and Pareschi (2021) PDE-ODE models on graphs are introduced to describe the spread of epidemics, by considering ODE compartment systems on the network nodes and PDE on edges. In literature, few works consider coupled PDE systems defined on different domains. Among these, we can cite the approach of noncoincident spatial domains (Fitzgibbon et al. 2004, 2005; Fitzgibbon and Langlais 2008; Fitzgibbon et al. 2022), the point-to-point coupling approach on identical or similar domains (Ambrosio and Aziz-Alaoui 2012; Ambrosio et al. 2019; Ambrosio and Aziz-Alaoui 2020; Aziz-Alaoui and Parimita 2021; Cantin et al. 2019; Cantin and Aziz-Alaoui 2021; Miranville et al. 2021), and the point-to-point approach with a corridor connecting domains (Aziz-Alaoui et al. 2024). Each of them have specific advantages depending on the characteristics of the studied physical system.

Our present work is inspired by a reaction-diffusion model with non-local coupling terms proposed in Freedman and Zhao (1999), for the dispersion of populations between distant islands. The evolution of a population in a specific domain is described by a PDE, with the possibility of migrating from within one domain to another, modeled via non-local terms. The non-local approach stands out for its unique ability to model long-distance interactions between spatially disjoint domains, offering increased flexibility in the shape, size and dimension of the domains.

In our paper we adopt a similar approach as in Freedman and Zhao (1999), by considering the case of *n* disjoint domains and taking into account also boundary-interior connections. We would like to emphasize that our work is not a merely extension of Freedman and Zhao (1999) to the case of *n* domains and *m* species. Indeed, our main aim is to establish an explicit and generalizable modeling framework.

With respect to Freedman and Zhao (1999), the pivotal contributions of our approach are outlined as follows:

- the clear separation of spatial variables specific to each domain, without particular assumptions on their geometry;
- the explicit definition and interpretation of incoming/outgoing flows and node-to-node connections, which are often left implicit in existing works (Ambrosio and Aziz-Alaoui 2012; Ambrosio et al. 2019; Ambrosio and Aziz-Alaoui 2020; Cantin et al. 2019; Cantin and Aziz-Alaoui 2021);
- the systematic integration of the flow conservation principle as an indicator of the structural consistency of the coupling.

Thus enabling to obtain an exact recovery of Freedman’s model (Freedman and Zhao 1999) in the two-node case, a consistent extension to *n* nodes with heterogeneous and boundary-interior migrations, and a general and explicit formalism for the construction of systems of PDE on networks.

For this type of coupled models it is also interesting to investigate the“transposition”of properties, from a node to the global network. In other words, if each node in the network (before coupling) has solutions sharing common properties such as existence, positivity, boundedness, periodicity, regularity, does the solution of the network model retain these same properties?

This article is divided into three sections. We present the construction of a coupled PDE network where a population evolves on each node. We introduce the necessary definitions and notations (nodes, edges, flow) to understand the coupled network model we propose. In this section, we demonstrate the principle of flow conservation and show the existence of a maximal positive solution to the coupled model.

In the second section, building on the notations and definitions established in the first part, we propose the formulation of the coupled model for a network where the evolution of multiple populations is modeled on each node. We demonstrate that flow conservation is always verified. Furthermore, in the case of networks where mass conservation occurs at each node, we prove that, in the coupled model, there is also mass conservation. We succeed in transposing to the entire network the property of uniform boundedness when it is verified on all nodes.

The third part consists of an application where we study a coupled prey-predator model on a network with two nodes, and provide numerical simulations to illustrate our results.

## A dynamical model on a network: scalar case and vectorial cases

### A network model for the scalar case

#### Building the model

We consider a network system composed of *n* reaction-diffusion equations, each describing the evolution of a population distributed in *n* (n≥2) distinct domains $\Omega _i$ ($i\in \Sigma =\{1,\dots , n\}$). We will denote by $u_i=u(t,x_i)$ the density of population in the domain $\Omega _i$. Here we denote by $x_i$ a generic point in $\Omega _i$. We suppose that the population can migrate from a domain to the others. Our objective is to develop a coupled model that faithfully captures the dynamics of the population in these *n* domains. In absence of migrations, the evolution of the population density on each domain is governed by the following equations:

1 $$\begin{aligned} {\left\{ \begin{array}{ll} \displaystyle \partial _t u_i(t,x) = D_i\Delta u_i(t,x_i) + F_i(u_i(t,x_i)), & t> 0, \quad x_i \in \Omega _i, \\ \displaystyle \partial _{\eta _i}u_i (t,x_i) = 0, & t > 0, \quad x_i \in \partial \Omega _i, \quad i \in \Sigma , \end{array}\right. } \end{aligned}$$

supplemented with some initial data $u_i(0,x_i)=u_i^0(x_i)$, whose properties will be specified below.

Within this framework, for each node, $i \in \{1, \ldots , n\},$

- the population density in $\Omega _i$ is denoted by
  2 $$\begin{aligned} u_i : \mathbb {R}^{+} \times \overline{\Omega }_i \;\longrightarrow \; \mathbb {R}. \end{aligned}$$
- Δ is the diffusion operator on $\Omega _i$, modeling the motion by random diffusion of the population inside one domain. Moreover $D_i > 0$ is the diffusion coefficient of the species *i* in $\Omega _i$.
- $F_i:\mathbb {R}\rightarrow \mathbb {R}$ is a $K_i$-Lipschitz fonction corresponding to the reaction term for the population evolving in the domain $\Omega _i$.
- The vector $\eta _i(x_i)$ denotes the unit outward normal vector of $\Omega _i$ at the boundary point of $x\in \partial \Omega _i$.

**Fig. 1:**
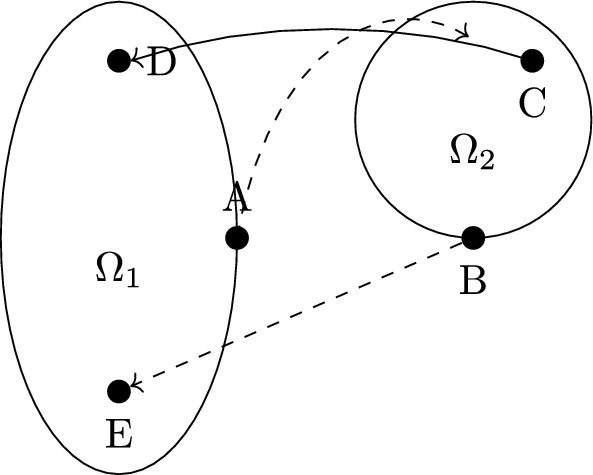
Types of connections between different domains. Internal connections are represented by solid lines and boundary connections by dashed lines.

Throughout the rest of this article, we consider the following hypotheses:

$(H_1)$: For all $i \in \Sigma$, we assume that $\Omega _i \subset \mathbb {R}^d$ is a nonempty, open, bounded and regular domain, for some dimension d≥1.
$(H_2)$: The closures of the domains $\Omega _i$ are pairwise disjoint, namely
  $$ \overline{\Omega }_i\cap \overline{\Omega }_j\ne \emptyset ,\;\;\forall i\ne j. $$
  *This hypothesis is used to situation where the domains are isolated, allowing for non-local mobility between the different domains, in addition to mobility within each domain.*
$(H_3)$: Two types of migrations are possible (see Figure 1)

1. **Internal migrations**: from $\Omega _i$ to $\Omega _j$.
2. **Boundary migrations**: from $\partial \Omega _i$ to $\Omega _j$.

*Our model accounts for movements from one isolated environment to another. As a special case, it applies to human migrations as well as the spread of invasive species, both animal and plant, particularly when occurring over long distances via transport modes such as ships, trains, or airplanes. This hypothesis implicitly assumes that migrations of the population towards boundaries (either from boundary to boundary or from the interior of a domain to a boundary of another domain) are not considered. Such migrations is not explored in this work. However this can be extended, using similar arguments as the one given in this note when migrations depends linearly of the population density, and using more refined arguments (extrapolation spaces*Lunardi (2012) *or integrated semigroups* Ducrot and Magal (2020)*) when these terms depend nonlinearly from the state variables.*

We now consider coupling between domains and proceed to formalize the population displacements between domains by introducing the concepts of inter-domain displacement probabilities, network edges, and network flows. These definitions are crucial for quantifying population movements between different spatial regions.

**Inter-domain displacement probability**

For each pair $(i,j) \in \Sigma ^2$, i≠j, we introduce the continuous function

$$ K_{ji} : \overline{ \Omega }_i \times \overline{ \Omega }_j \longrightarrow [0, 1], $$

which describes the probability from moving from $x_i\in \overline{\Omega }_i$ to $x_j\in \Omega _j$. For any point $x_i \in \overline{\Omega }_i$, we also denote by $P_{ji}(x_i)$ the probability of moving from the point $x_i$ to the domain $\Omega _j$, this can be rewritten as

3 $$\begin{aligned} P_{ji}(x_i):=\int _{\Omega _j}K_{ji}(x_i,y)dy,\;\;x_i\in \overline{\Omega }_i. \end{aligned}$$

**Network edges**

In the following we will say that the domain $\Omega _i$ is connected to the domain $\Omega _j$, with i≠j, if migration of a population from one domain to the other may occur, that is if there exists $x_i\in \overline{\Omega }_i$ such that $P_{ji}(x_i)\ne 0$.

This approach leads us to the construction of a network. Here each node *i* represents a domain $\Omega _i$, while an edge from *i* to *j* means that there is a migration of population from the domain $\Omega _i$ to the domain $\Omega _j$. Since we have supposed two different types of migration (internal migrations: from inside to inside and boundary migrations: from the boundary to inside), we consider two types of edges:

- Internal Edges (that comes from internal migrations)
  4 $$\begin{aligned} E_I:=\left\{ (i,j) \in \Sigma ^2, i\ne j, \ \vert \ \exists \ x_i \in \Omega _i \ \vert \ P_{ji}(x_i)\ne 0 \right\} . \end{aligned}$$
- Boundary Edges (taking into account the boundary migrations)
  5 $$\begin{aligned} E_B:=\left\{ (i,j) \in \Sigma ^2, i\ne j, \ \vert \ \exists \ x_i \in \partial \Omega _i \ \vert \ P_{ji}(x_i)\ne 0 \right\} . \end{aligned}$$

Moreover, for all $i \in \Sigma$, we define

- $\displaystyle I_i^{out}:=\left\{ j \in \Sigma \backslash \{i\} \ \vert \ (i,j) \in E_I \right\}$, the set of indices of domains connected to domain $\Omega _i$ by outgoing internal connections.
- $\displaystyle I_i^{in}:=\left\{ j \in \Sigma \backslash \{i\} \ \vert \ (j,i) \in E_I \right\}$, the set of indices of domains connected to domain $\Omega _i$ by incoming internal connections.
- $\displaystyle B_i^{out}:=\left\{ j \in \Sigma \backslash \{i\} \ \vert \ (i,j) \in E_B \right\}$, the set of indices of domains connected to domain $\Omega _i$ by outgoing boundary connections.
- $\displaystyle B_i^{in}:=\left\{ j \in \Sigma \backslash \{i\} \ \vert \ (j,i) \in E_B \right\}$, the set of indices of domains connected to domain $\Omega _i$ by incoming boundary connections.

##### Proposition 2.1

(Structure of edges) The sets of boundary edges $E_B$ and interior edges $E_I$ satisfy the following properties:

$$\begin{aligned} E_B=\bigcup _{k \in \Sigma } \{k\}\times B_k^{out}=\bigcup _{k \in \Sigma } B_k^{in}\times \{k\} \qquad E_I=\bigcup _{k \in \Sigma }\{k\}\times I_k^{out}=\bigcup _{k \in \Sigma } I_k^{in} \times \{k\} . \end{aligned}$$

##### Proof

The first equality for $E_B$ is immediate from the definition of $B_k^{out}$. To show the second, let $(i,j)\in E_B$. Then $i\in B_j^{in}$ and $j\in \Sigma$, so $(i,j)\in \bigcup _{k\in \Sigma } B_k^{in}\times \{k\}$. The reverse inclusion is clear, thus equality holds. Similarly, one shows the equalities for $E_I$. □

**Network coupled model**:

After having defined the network structure we are now able to introduce our system on network ($i\in \Sigma$):

6 $$\begin{aligned} \begin{aligned} {\left\{ \begin{array}{ll} \displaystyle \partial _t u_i (t,x_i)& = D_i\Delta u_i(t,x_i) + F_i(u_i(t,x_i)) -\displaystyle \sum _{j \in I_i^{out}}\varphi _{ji}(u_i(t,x_i))P_{ji}(x_i)\\ & \hspace{0.5cm}+\displaystyle \varepsilon _i(x_i)\sum _{j \in I_i^{in}}\int _{\Omega _j}\varphi _{ij}(u_j(t,x_j))P_{ij}(x_j)dx_j \displaystyle \\ & \hspace{0.5cm}+ \displaystyle \varepsilon _i(x_i)\sum _{j \in B_i^{in}}\int _{\partial \Omega _j}g_{ij}(u_j(t,x_j))P_{ij}(x_j)d\sigma \left( x_j\right) , \; t \ge 0, \ x_i \in \Omega _i, \\ \displaystyle \partial _{\eta _i}u_i(t,x_i)& =\displaystyle -\sum _{j \in B_i^{out}}g_{ji}(u_i(t,x_i))P_{ji}(x_i), \qquad \qquad \qquad t \ge 0, \ x_i \in \partial \Omega _i. \end{array}\right. } \end{aligned} \end{aligned}$$

Each node $i\in \Sigma$ has its own internal dynamics defined as in (1) and the supplementary terms in (6) model the different migrations among the domains. In particular:

- the quantity $\varphi _{ji}(u_i(t,x_i))P_{ji}(x_i)$ represents the outcoming flow of individuals leaving a point $x_i \in \Omega _i$ towards the domains $\Omega _j$, with j≠i. The term $\varphi _{ji}(u_i(t,x_i))$ models the maximal number of individuals that at time t>0 can migrate from $x_i\in \Omega _i$ to $\Omega _j$. Thus,
  $$\begin{aligned} \displaystyle \sum _{j \in I_i^{out}}\varphi _{ji}(u_i(t,x_j))P_{ji}(x_i) \end{aligned}$$
  represents the total outcoming flow from $x_i\in \Omega _i$ with respect to internal migrations.
- the quantity $g_{ji}(u_i(t,x_i))P_{ji}(x_i)$ represents the outcoming flow of individuals leaving a point $x_i \in \partial \Omega _i$ and moving towards the domain $\Omega _j$, with j≠i. The term $g_{ji}(u_i(t,x_i)))$ models the maximal number of individuals that at time t>0 can migrate from $x_i\in \partial \Omega _i$ to $\Omega _j$. Thus,
  $$\begin{aligned} \displaystyle \sum _{j \in B_i^{out}}g_{ji}(u_i(t,x_j))P_{ji}(x_i) \end{aligned}$$
  represents the total outcoming flow from $x_i\in \partial \Omega _i$ with respect to boundary migrations.
- the quantity $\int _{\Omega _j}\varphi _{ij}(u_j(t,x_j))P_{ij}(x_j)dx_j$ describes the number of individuals that leave the domain $\Omega _j$ to move to $\Omega _i$. It is worth noting that
  $$\ \int _{\Omega _j}\varphi _{ij}(u_j(t,x_j))P_{ij}(x_j)dx_j = \int _{\Omega _j}\varphi _{ij}(u_j(t,x_j))\int _{\Omega _i}K_{ji}(x_j,y)dydx_j $$
  does not depend on $x_i\in \Omega _i$. Indeed, the distribution in $\Omega _i$ of the individuals migrating from the other domains is defined through the function $\varepsilon _i \in \mathcal {C}^{0}\left( \overline{\Omega }_i,[0,1]\right)$ that models the probability of receiving migrants at location $x_i \in \Omega _i$. Thus, we can conclude that the term
  $$\begin{aligned} \displaystyle \varepsilon _i(x_i)\sum _{j \in I_i^{in}}\int _{\Omega _j}\varphi _{ij}(u_j(t,x_j))P_{ij}(x_j)dx_j \end{aligned}$$
  represents the total incoming flow towards $x_i\in \Omega _i$ with respect to internal migrations.
- in the same way we can define the total incoming flow of individuals coming to $x_i\in \Omega _i$ from the boundaries of the other domains as
  $$\begin{aligned} \displaystyle \varepsilon _i(x_i)\sum _{j \in B_i^{in}}\int _{\partial \Omega _j}g_{ij}(u_j(t,x_j))P_{ij}(x_j)d\sigma \left( x_j\right) . \end{aligned}$$

In the sequel, for all $i \in \Sigma$, $j \in I_{i}^{out}$, and $k \in B_{i}^{out}$, let us consider $\varphi _{ji}$ and $g_{ki}$ linear functions of the density $u_i$:

$$\begin{aligned} \varphi _{ji}(u_i) = \alpha _{ji}u_i, \quad \text {and} \quad g_{ki}(u_i) = \tilde{\alpha }_{ki}u_i \end{aligned}$$

with

$$\begin{aligned} 0 \le \alpha _{ji} \le 1, \quad 0 \le \sum _{j \in I_i^{out}} \alpha _{ji} \le 1, \nonumber \\ 0 \le \tilde{\alpha }_{ji} \le 1, \quad 0 \le \sum _{k \in B_i^{out}} \tilde{\alpha }_{ki} \le 1 \end{aligned}$$

Moreover, let us introduce

$$\begin{aligned} m_{ji}(x_i)&=\alpha _{ji}P_{ji}(x_i) \quad \text {and}\qquad m_i(x_i) = \sum _{j \in I_i^{out}} m_{ji}(x_i) \qquad \forall \, x_i \in \Omega _i,\\ \tilde{m}_{ji}(x_i)&=\tilde{\alpha }_{ji}P_{ji}(x_i) \quad \text {and}\qquad \tilde{m}_i(x_i) = \sum _{j \in I_i^{out}} \tilde{m}_{ji}(x_i) \qquad \forall \, x_i \in \partial \Omega _i. \end{aligned}$$

and let us suppose that

$$\begin{aligned} 0&\le \sum _{j \in I_i^{out}} \alpha _{ji}P_{ji}(x_i) \le 1, \quad \forall x_i \in \Omega _i\\ 0&\le \sum _{k \in B_i^{out}} \tilde{\alpha }_{ki}P_{ki}(x_i) \le 1, \quad \forall x_i \in \partial \Omega _i. \end{aligned}$$

##### Remark 2.3

The choice of the form of functions $\varphi _{ji}$ and $g_{ki}$, with $i \in \Sigma$, $j \in I_i^{\textrm{out}}$ and $k \in B_i^{\textrm{out}}$, has been adopted mainly for notational convenience in the subsequent analysis and because it is reasonable in light of the intended applications. In particular, linear functions were chosen in order to model the outgoing population fluxes from the domain $\Omega _i$. Nonetheless, the properties of the model established hereinafter continue to hold for more general classes of functions. More precisely, the analysis extends to nonlinear functions that are positive, at least continuous, and bounded.

Thus, model (6) reads as follows, for $i\in \{1,..,n\}$:

7 $$\begin{aligned} \hspace{-0.5cm} \left\{ \begin{aligned} \partial _t u_i(t,x_i)&= D_i \Delta u_i + F_i(u_i(t,x_i)) - m_i(x_i)u_i(t,x_i) \\&\quad + \varepsilon _i(x_i)\sum _{j \in I_i^{\textrm{in}}} \int _{\Omega _j} u_j(t,x_j)m_{ij}(x_j)\,dx_j \\&\quad + \varepsilon _i(x_i)\sum _{j \in B_i^{\textrm{in}}} \int _{\partial \Omega _j} u_j(t,x_j)\tilde{m}_{ij}(x_j)\,d\sigma (x_j),&\quad&t \ge 0,\; x_i \in \Omega _i, \\ \partial _{\eta _i} u_i(t,x_i)&= -\tilde{m}_i(x_i)u_i(t,x_i),&\quad&t \ge 0,\; x_i \in \partial \Omega _i, \\ u_i(0,x_i)&= u_{i,0}(x_i),&\quad&x_i \in \overline{\Omega }_i. \end{aligned} \right. \end{aligned}$$

##### Remark 2.4

We assume here that the movement between patches, and the location at which members of a population may arrive after a transition, depends only on the spatial location, but not on the density itself, even if this question is interesting.

It is indeed natural to assume that people will avoid crowds. Such a question would lead to nonlinear transitions depending on density, a problem that will be addressed in a future study.

##### Remark 2.5

We refer to non-local coupling because the construction of the flows defined previously takes into account the possibility of sending and receiving flow from any point within the domain. This non-local interaction is modeled by an integral evaluated over the entire domain. This is one of the strengths of this method, as it avoids making additional assumptions about the domains, as often observed in the literature.

##### Remark 2.8

This model generalizes and improves on the 2-nodes model proposed in Freedman and Zhao (1999), since it is specifically designed for a network of finite size greater than or equal to 2. Indeed, our model incorporates not only internal migrations but also boundary ones. The inclusion of boundary migrations brings several advantages, including a better representation of realistic interactions between domains, a more precise analysis of population flows at boundaries, and an increased understanding of migration dynamics in various contexts, significantly enriching the application and relevance of the model.

#### Model analysis: Well posedness and conservation of flow

Under the above set of hypotheses, we will obtain the following well-posedness and positivity of solution for (7).

##### Proposition 2.6

For each initial data $(u_{1,0},\cdots ,u_{n,0})\in \mathcal {C}^0\left( \overline{\Omega }_1,\mathbb {R}^+\right) \times \cdots \times \mathcal {C}^0\left( \overline{\Omega }_n,\mathbb {R}^+\right)$, there exists a maximum time of existence $T_\textrm{max}>0$ and *n* non-negative functions $u_1=u_1(t,x_1)$, ...,$u_n=u_n(t,x_n)$ defined and continuous on $[0,T_\textrm{max})\times \overline{\Omega }_i$, such that $(u_1,\cdots u_n)$ is a mild solution of (7), in the sense of semigroups as described in the appendix below. Furthermore $T_\textrm{max}$ is maximal in the sense that either $T_\textrm{max}=\infty$ or $T_\textrm{max}<\infty$ and

$$ \max _{i=1,..,n}\sup _{x_i\in \Omega _i} u(t,x_i)\rightarrow \infty \text { as }t\nearrow T_\textrm{max}. $$

This result will be proved in the proof 1. This result will be obtained as a special case of a more general framework where, various species can live and interact with other species in each domain $\Omega _i$ and possibly moves from one set to another.

The principle of flow conservation states that the total flow of individuals moving between different domains remains constant. In practical terms, this means that, at any given moment, the total number of individuals entering and leaving each domain balances perfectly, resulting in a net zero flow. In other words, any individual leaving one domain enters simultaneously in another domain, ensuring that there is neither a loss nor a gain of individuals during the transit. This concept is essential to ensure that our model faithfully mimics closed systems, where population movements occur without the sudden creation or disappearance of individuals.

##### Proposition 2.7

(Flow conservation) For all $n \in \mathbb {N}$, n≥2, the model (7) defined on the network $G_n=(V,E)$ with $V = \{\overline{\Omega }_i, \ i \in \Sigma \}$ and $E=E_I \cup E_B$ satisfies the principle of flow conservation, namely

$$ \sum _{i \in \Sigma } S_i(u,t) = 0, $$

where $u=(u_1,\cdots u_n)$ is a solution of (7) and

8 $$\begin{aligned} \begin{aligned} S_i(u,t) = \int _{\Omega _i}&\varepsilon _i(x_i) \sum _{j \in I_i^{in}}\int _{\Omega _j}\varphi _{ij}(u_j(t,x_j))P_{ij}(x_j)dx_j dx_i \\&+ \int _{\Omega _i}\varepsilon _i(x_i)\sum _{j \in B_i^{in}}\int _{\partial \Omega _j}g_{ij}(u_j(t,x_j))P_{ij}(x_j)d\sigma \left( x_j\right) dx_i\\&- \int _{\Omega _i}\sum _{j \in I_i^{out}}\varphi _{ji}(u_i(t,x_j))P_{ji}(x_i)dx_i \\&- \int _{\partial \Omega _i}\sum _{j \in B_i^{out}}g_{ji}(u_i(t,x_i))P_{ji}(x_i)d\sigma \left( x_i\right) . \end{aligned} \end{aligned}$$

##### Proof

By Proposition 2.1, the sums over incoming and outgoing edges can be reorganized. All incoming flows are exactly balanced by outgoing flows:

$$ \sum _{i \in \Sigma } S_i(u,t) = 0. $$

This proves that the total flow is conserved at any time *t*. □

##### Remark 2.9

Integrating (7) over each domain $\Omega _i$ and summing over $i \in \Sigma$ gives

$$ \frac{d}{dt} \left( \sum _{i=1}^n \int _{\Omega _i} u_i(t,x_i)\, dx_i \right) = \sum _{i=1}^n \int _{\Omega _i} F_i(u_i)\, dx_i. $$

In particular, in the absence of birth and deaths process ($F_i = 0$) on each domain, the total mass is conserved. Thus, flow conservation of the flow and local mass conservation directly imply conservation of the total population over the network.

##### Remark 2.2

Under the assumption that each reaction function $F_i$ is at most linear, i.e., there exist constants $C_i^1>0$ and $C_i^2>0$ such that

$$ |F_i(u_i)| \le C_i^1 |u_i| + C_i^2, \quad \forall u_i \in \mathbb {R}, $$

the local solution obtained in Proposition 2.6 can be extended to a global solution defined for all t≥0 (see Corollary 5.3.4 in Magal and Ruan (2018)). In other words, $u_i(t,x_i)$ remains bounded on $\overline{\Omega }_i$ for each domain $\Omega _i$, thus ensuring the global existence of the coupled system on the network.

This property relies on the fact that linear growth prevents finite-time blow-up of the solution. The coupling terms, being proportional to the local densities, do not alter this bound, thereby guaranteeing global control of the population.

### A dynamical model on a network: vectorial case

#### The coupled model

In this section, in contrast to the model described in the previous section where each network node is assumed to contain a single population (scalar case), we extend the model to the case where in each domain several populations (m≥2) are interacting (vector case); that is, each domain, symbolized by $\Omega _i$ ($i \in \Sigma = \{1, \dots , n\}$), hosts *m* distinct subpopulations.

Within the domain $\Omega _i$, each subpopulation is characterized by its density $U_i^k$ (k=1,⋯,m). Thus, the set of densities of subpopulations in a given domain $\Omega _i$ is represented by $U_i = \left( U_i^k\right) _{1 \le k \le m}$. The dynamics of each subpopulation are governed by a system of specific reaction-diffusion equations, capturing the mechanisms of birth, death, interactions within the population densities as well as the spatial movement to each group.

To illustrate the relevance of this model, let us consider a continental scale as an example. Imagine a continent where each nation or region constitutes a distinct and interconnected domain $\Omega _i$. The total population of the continent is distributed among these entities, with each country hosting a specific fraction of the population. Within each country, the population can be further divided into subgroups based on demographic characteristics such as age, gender or nationality. The dynamics of each subpopulation are regulated by adapted reaction-diffusion equations, taking into account parameters such as birth rates, mortality rates, and migratory movements, which vary from one country to another.

For each $i \in \{1,..,n\}$, in the absence of migration, the dynamics on the single node $\Omega _i$ is described by the densities of each population, denoted by $U_i^k=U_i^k(t,x_i)$ for k=1,..,m, and satisfying the following system of equations,

9 $$\begin{aligned} {\left\{ \begin{array}{ll} \displaystyle \partial _t U_i^1 = D_i^1 \Delta U_i^1 + F_i^1(U_i), \quad & t> 0, \ x_i \in \Omega _i \\ \vdots \\ \displaystyle \partial _t U_i^m = D_i^m \Delta _{i} U_i^m + F_i^m(U_i), \quad & t> 0, \ x_i \in \Omega _i \\ \partial _{\eta _i} U_i^1 =0, \quad & t> 0, \ x_i \in \partial \Omega _i,\\ \vdots \\ \partial _{\eta _i} U_i^m=0, \quad & t > 0, \ x_i \in \partial \Omega _i, \\ U_i^1(0,x_i) = U_{i,0}^1(x_i) \quad & x_i \in \overline{\Omega }_i,\\ \vdots \\ U_i^m(0,x_i) = U_{i,0}^m(x_i) \quad & x_i \in \overline{\Omega }_i.\\ \end{array}\right. } \end{aligned}$$

Here recall that $U_i^k$ denotes the $k^{th}$ component of the vector $U_i$, $D_i^k$ represents the diffusion coefficient of $U_i^k$ in $\Omega _i$ while $F_i^k(U_i)$ is the reaction term for subpopulation *k* in $\Omega _i$. In the following, system (9) will be referred to as the $i^{th}$ subsystem.

By introducing migration between domains, we add the following hypothesis:

$(H_4)$: For each pair (*i*, *j*) belonging to the set $E_I \cup E_B$ and for all $k \in \{1, \ldots , m\}$, if a proportion of subpopulation $U_i^k$ migrates to domain $\Omega _j$, then the migratory flow induced by the portion of individuals $U_i^k$ contributes to the growth of subpopulation $U_j^k$ in domain $\Omega _j$. In this way, it is assumed that the exchange of flows between domains respects the type of individual that has migrated (for example, the prey species retains its role as prey, while the predator species retains its role as predator).

Therefore, we can now give the extending model:

10 $$\begin{aligned} \hspace{-0.6cm} {\left\{ \begin{array}{ll} \displaystyle \partial _t U_i^1 = D_i^1 \Delta U_i^1 + F_i^1(U_i)- m_i^1(x_i) \ U_i^1 +\theta _i^1(U(t,.),x_i) \, & t> 0, \ x_i \in \Omega _i \\ \vdots \\ \displaystyle \partial _t U_i^m = D_i^m \Delta _{i} U_i^m + F_i^m(U_i)- m_i^m(x_i) \ U_i^m +\theta _i^m(U(t,.),x_i) \, & t> 0, \ x_i \in \Omega _i \\ \partial _{\eta _i} U_i^1 = -\tilde{m}_i^1(x_i) \ U_i^1 \, & t> 0, \ x_i \in \partial \Omega _i\\ \vdots \\ \partial _{\eta _i} U_i^m=-\tilde{m}_i^m(x_i) \ U_i^m \, & t > 0, \ x_i \in \partial \Omega _i \\ U_i^1(0,x_i) = U_{i,0}^1(x_i) \, & x_i \in \Omega _i\\ \vdots \\ U_i^m(0,x_i) = U_{i,0}^m(x_i) \, & x_i \in \Omega _i,\\ \end{array}\right. } \end{aligned}$$

wherein we have set $U =\left( U_i\right) _{i \in \Sigma }^T$, with $U_i=U_i(t,x_i)=\left( U_i^k(t,x_i)\right) _{1\le k \le m}$ while functions $m_i^k$ and $\tilde{m}_i^k$ are given by

$$ m_i^k(x) =\sum \limits _{{j \in I_i^{out}}} m_{ji}^k(x), \ \tilde{m}_i^k(y)=\sum \limits _{{j \in B_i^{out}}} \tilde{m}_{ji}^k(y), $$

and the functions $\theta _i^k$ are given by

$$\begin{aligned} \theta _i^k(U(t,.),x_i) =\varepsilon _i^{k}(x_i)&\left( \sum \limits _{{j \in I_i^{in}}}^{} \int _{\Omega _j}m_{ij}^k(x_j)U_j^k(t,x_j)dx_j\right. \\&\left. +\sum \limits _{{j \in B_i^{in}}}^{}\int _{\partial \Omega _j}\tilde{m}_{ij}^k(x_j)U_j^k(t,x_j)d\sigma \left( x_j\right) \right) , \end{aligned}$$

where

- $\varepsilon _i^{k}(x_i)$ represents the probability for the *k*-th species of being hosted in $x_i\in \Omega _i$.
- $m_{ji}^k(.)= \alpha _{ji}^k \ P_{ji}^{k}(.)$ and $\tilde{m}_{ji}^k(.) = \tilde{\alpha }_{ji}^k \ P_{ji}^k(.)$ Here $\alpha _{ji}^k\in \mathbb {R}_+$ represents the maximum proportion of individuals $U_i^k$ that can migrate from $\Omega _i$ to $\Omega _j$, $\tilde{\alpha }_{ji}^k\in \mathbb {R}_+$ is the maximum proportion of individuals $U_i^k$ that can migrate from the boundary of domain $\Omega _i$ to $\Omega _j$, and $P_{ji}^k(.)$ is the probability for individuals of the *k*-th species to move from $\Omega _i$ to $\Omega _j$.

Thus, one obtains a model of n×m equations and *n* spatial variables $x_i$, each corresponding to one of the *n* spatial domains considered.

For example, in the case of n=2, m=2, and a bidirectional coupling concerning both internal and boundary migrations, we get the following set of equations:

$$\begin{aligned} {\left\{ \begin{array}{ll} {\left\{ \begin{array}{ll} \displaystyle \partial _t U_1^1 = D_1^1 \Delta U_1^1 + F_1^1(U_1^1,U_1^2)- m_{21}^1(x_1) \ U_1^1 +\theta _1^1(U,x_1)\quad & t> 0, \ x_1 \in \Omega _1 \\ \displaystyle \partial _t U_1^2 = D_1^2 \Delta _{1} U_1^2 + F_1^2(U_1^1,U_1^2)- m_{21}^2(x_1) \ U_1^2 +\theta _1^2(U,x_1) \quad & t> 0, \ x_1 \in \Omega _1 \\ \partial _{\eta _1} U_1^1 = -\tilde{m}_{21}^1(x_1) \ U_1^1 \quad & t> 0, \ x_1 \in \partial \Omega _1 \\ \partial _{\eta _1} U_1^2= -\tilde{m}_{21}^2(x_1) \ U_1^2 \quad & t> 0, \ x_1 \in \partial \Omega _1 \\ U_1^1(0,x_1) = U_{1,0}^1(x_1) \quad & x_1 \in \Omega _1\\ U_1^2(0,x_1) = U_{1,0}^2(x_1) \quad & x_1 \in \Omega _1\\ \end{array}\right. } \\ {\left\{ \begin{array}{ll} \displaystyle \partial _t U_2^1 = D_2^1 \Delta U_2^1 + F_2^1(U_2^1,U_2^2)- m_{12}^1(x_2) \ U_2^1 +\theta _2^1(U,x_2) \quad & t> 0, \ x_2 \in \Omega _1 \\ \displaystyle \partial _t U_2^2 = D_2^2 \Delta _{1} U_2^2 + F_2^2(U_2^1,U_2^2)- m_{12}^2(x_2) \ U_2^2 +\theta _2^2(U,x_2) \quad & t> 0, \ x_2 \in \Omega _2 \\ \partial _{\eta _2} U_2^1 = -\tilde{m}_{12}^1(x_2) \ U_2^1 \quad & t> 0, \ x_2 \in \partial \Omega _2 \\ \partial _{\eta _2} U_2^2= -\tilde{m}_{12}^2(x_2), \ U_2^2 \quad & t > 0, \ x_2 \in \partial \Omega _2 \\ U_2^1(0,x_2) = U_{2,0}^1(x_2) \quad & x_2 \in \Omega _2\\ U_2^2(0,x_2) = U_{2,0}^2(x_2) \quad & x_2 \in \Omega _2,\\ \end{array}\right. } \end{array}\right. } \end{aligned}$$

where $$U=\left( U_1^1,U_1^2,U_2^1,U_2^2\right) ^T$$ and

$$\begin{aligned}&\theta _1^1(U,x_1)=\varepsilon _1^1(x_1)\left( \int _{\Omega _2}m_{12}^1(x_2)U_2^1(t,x_2)dx_2 \displaystyle +\int _{\partial \Omega _2}\tilde{m}_{12}^1(x_2)U_2^1(t,x_2)dx_2\right) \\&\theta _1^2(U,x_1)=\varepsilon _1^2(x_1)\left( \int _{\Omega _2}m_{12}^2(x_2)U_2^2(t,x_2)dx_2 \displaystyle +\int _{\partial \Omega _2}\tilde{m}_{12}^2(x_2)U_2^2(t,x_2)dx_2\right) \\&\theta _2^1(U,x_2)=\varepsilon _2^1(x_2)\left( \int _{\Omega _1}m_{21}^1(x_1)U_1^1(t,x_1)dx_1 \displaystyle +\int _{\partial \Omega _1}\tilde{m}_{21}^1(x_1)U_1^1(t,x_1)dx_1\right) \\&\theta _2^2(U,x_2)=\varepsilon _2^2(x_2)\left( \int _{\Omega _1}m_{21}^2(x_1)U_1^2(t,x_1)dx_1 \displaystyle +\int _{\partial \Omega _1}\tilde{m}_{21}^2(x_1)U_1^2(t,x_1)dx_1\right) . \end{aligned}$$

#### Model properties: Well-posedness, flow conservation and mass conservation

The coupled system (10) can be formally rewritten in the framework of an abstract Cauchy problem. For the sake of brevity, we refer the reader to Appendix A for the precise functional setting and detailed derivation. In this abstract formulation, the system is expressed as

$$\begin{aligned} \frac{dU(t)}{dt} = A U(t) + G(U(t)), \quad t>0, \quad U(0) = U_0 \in X_+, \end{aligned}$$

where *A* is a linear operator generating a strongly continuous and positive semigroup, *G* is a nonlinear map encoding the local interactions and the nonlocal migration between domains, and $X_+=X_{1,+}\times \cdots \times X_{n,+}$, with $X_{i,+}:=\left( \mathcal {C}\left( \overline{\Omega }_i; \mathbb {R}^+\right) \right) ^m$ $(i\in \Sigma )$.

Under the assumptions detailed in Appendix A, we have the following result:

##### Proposition 2.10

For all initial data $U_0 \in X_+$, there exists a maximal existence time $T_\textrm{max}>0$ and a unique function

$$ U \in \mathcal {C}([0,T_\textrm{max}); X_+) $$

such that

11 $$\begin{aligned} U(t) = T_A(t) U_0 + \int _0^t T_A(t-s) G(U(s)) ds, \quad \forall t \in [0, T_\textrm{max}). \end{aligned}$$

Moreover, either $T_\textrm{max} = \infty$, or $T_\textrm{max} < \infty$ and

$$ \lim _{t \nearrow T_\textrm{max}} \Vert U(t)\Vert _X = \infty . $$

The proof of this result relies on standard semigroup theory and the local Lipschitz continuity of *G* (see Theorem 1.4 in Pazy (1983)), while positivity of the solution follows from Proposition 5.1 in Magal and Ruan (2018).

Let *U* be a solution of (10). The principle of flow conservation for model (10) is expressed as follows, for all $k\in \{1,\dots m\}$:

12 $$\begin{aligned} \sum _{i \in \Sigma } \int _{\Omega _i} (\theta _i^k(U(t,.),x_i) - m_i^k(x_i) U_i^k) dx_i - \int _{\partial \Omega _i} \tilde{m}_i^k(x_i) U_i^k d\sigma (x_i) = 0 \end{aligned}$$

##### Proposition 2.11

For all $n, \ m \in \mathbb {N}$ with n,m≥2, let us consider $G_{n\times m} = (V, E)$, with $V = \left\{ \overline{\Omega }_i, \ i \in \Sigma \right\}$ and $E = E_I \cup E_B$ a network modeling the coupling of *n* reaction-diffusion equation systems of size *m* over *n* disjoint domains, as in (10). Under all the above hypotheses, then the principle of flow conservation is satisfied and we also have

13 $$\begin{aligned} \dfrac{d}{dt}\left( \sum _{i \in \Sigma }\int _{\Omega _i}U_i^k(t,x_i)dx_i\right) _{1\le k \le m}=\left( \sum _{i \in \Sigma }\int _{\Omega _i}F_i^k(U_i)dx_i\right) _{1\le k \le m} \end{aligned}$$

As for the scalar case, it is possible to see that, if each isolated system in (9) exhibits mass conservation, that is if

$$\begin{aligned} \dfrac{d}{dt}\left( \sum _{k =1}^m\int _{\Omega _i}U_i^k(t,x_i)dx_i\right) =0, \quad \forall \,i\in \Sigma , \end{aligned}$$

then the total mass conservation in the network is a natural consequence of the flow conservation:

##### Proposition 2.12

Under the above set of hypotheses, the coupled model (10) satisfies:

1. If for all $i \in \Sigma$, the operator $F_i(U_i)$ is quasi-positive, then the operator
  $$\begin{aligned} F_i(U_i)-m_i(x_i) \ U_i +\theta _i(U(t,.),.) \end{aligned}$$
  is also quasi-positive;
2. If for all $i \in \Sigma$, system (9) satisfies the principle of mass conservation, then network system (10) also satisfies this principle:
  $$\begin{aligned} \dfrac{d}{dt}\left( \sum _{k=1 }^m\sum _{i \in \Sigma }\int _{\Omega _i}U_i^k(t,x_i)dx_i\right) =0. \end{aligned}$$

## Case test: a predator-prey model on network

Let us consider a classical diffusive predator-prey model, defined by the following system of partial differential equations:

14 $$\begin{aligned} {\left\{ \begin{array}{ll} \partial _t u = D_u \Delta u + u(1-u) - \alpha uv,\\ \partial _t v = D_v \Delta v + \alpha uv - \mu v, \end{array}\right. } \end{aligned}$$

where *u* and *v* represent the prey and predator populations, respectively, $D_u >0$ and $D_v>0$ are diffusion coefficients, u(1-u) describes logistic growth of the prey, $\alpha uv \ge 0$ is the predation term, and $\mu v \ge 0$ represents predator mortality. The inclusion of diffusion terms models the spatial movement of the populations, making the model more realistic in extended environments.

### Nonlocal coupled two habitats model

We now consider two distinct habitats, $\Omega _1$ and $\Omega _2$, each hosting a prey-predator population $(u_i,v_i)$, $i\in \{1,2\}$. Migration between these two domains is modeled by the system:

15 $$\begin{aligned} {\left\{ \begin{array}{ll} \partial _t u_1 = D_{u_1} \Delta u_1 + F_{u_1}(u_1, v_1) - \alpha _{21} u_1 p_{21}(x) + \displaystyle \varepsilon _1(x) \alpha _{12} \int _{\Omega _2} u_2 p_{12}(y) dy,\\ \partial _t v_1 = D_{v_1} \Delta v_1 + F_{v_1}(u_1, v_1) - \alpha _{21} v_1 p_{21}(x) + \displaystyle \varepsilon _1(x) \alpha _{12} \int _{\Omega _2} v_2 p_{12}(y) dy,\\ \partial _{\eta _1} u_1 = \partial _{\eta _1} v_1 = 0, \quad t>0, \ x \in \partial \Omega _1,\\ \partial _t u_2 = D_{u_2} \Delta u_2 + F_{u_2}(u_2, v_2) - \alpha _{12} u_2 p_{12}(y) + \displaystyle \varepsilon _2(y) \alpha _{21} \int _{\Omega _1} u_1 p_{21}(x) dx,\\ \partial _t v_2 = D_{v_2} \Delta v_2 + F_{v_2}(u_2, v_2) - \alpha _{12} v_2 p_{12}(y) + \displaystyle \varepsilon _2(y) \alpha _{21} \int _{\Omega _1} v_1 p_{21}(x) dx,\\ \partial _{\eta _2} u_2 = \partial _{\eta _2} v_2 = 0, \quad t>0, \ y \in \partial \Omega _2, \end{array}\right. } \end{aligned}$$

with

$$ \begin{aligned} F_{u_1}(u_1,v_1)&= u_1(1-u_1) - \alpha _1 u_1 v_1,&F_{v_1}(u_1,v_1)&= \alpha _1 u_1 v_1 - \mu _1 v_1, \\ F_{u_2}(u_2,v_2)&= u_2(1-u_2) - \alpha _2 u_2 v_2,&F_{v_2}(u_2,v_2)&= \alpha _2 u_2 v_2 - \mu _2 v_2. \end{aligned} $$

The nonlocal coupling terms

$$ - \alpha _{ji} u_i p_{ji}(.) + \varepsilon _i(.) \alpha _{ij} \int _{\Omega _j} u_j p_{ij}(y) dy, \quad i\ne j $$

represent the migration of populations between habitats. The functions $p_{ij}$ (with $(i,j)\in \{1,2\}^2, i\ne j$) give the migration probabilities between the domains, $\alpha _{ij}$ determines the maximal flow of migrants, and $\varepsilon _i$ is the acceptance probability of migrants in domain $\Omega _i$. Here, we assume that these probabilities are independent of the species type. Moreover, only interior migrations are taken into account.

#### Theoretical analysis of the two-habitats network model

For each domain $i \in \{1,2\}$, we set

$$ U_i = (u_i, v_i), \quad F_i(U_i) = \begin{pmatrix} u_i(1-u_i) - \alpha _i u_i v_i \\ \alpha _i u_i v_i - \mu _i v_i \end{pmatrix}. $$

Each $F_i$ is a quadratic polynomial in $(u_i,v_i)$ and therefore locally Lipschitz, satisfying the main hypothesis of Proposition 2.10. By summing the components in each domain, it yields:

$$ F_i^1(U_i) + F_i^2(U_i) = u_i(1-u_i) - \mu _i v_i \le M_i^1 - M_i^2 (u_i + v_i), $$

with $M_i^1 = (1+\mu _i)/2$ and $M_i^2 = \mu _i$. This ensures that the total growth $u_i+v_i$ is bounded, guaranteeing the dissipativity of the system and allowing the application of Proposition 2.12. The nonlocal coupling terms are positive and bounded, ensuring quasi-positivity of the operator *G* of the coupled system. Therefore, if $U_0 \in X^+$, the solution remains positive for all t>0. Moreover, the system satisfies the flow conservation principle (Proposition 2.11), ensuring a consistent balance of populations between domains. We define the linear operator

$$ A := \begin{pmatrix} D_{u_1} \Delta _{\Omega _1}^N & 0 & 0 & 0 \\ 0 & D_{v_1} \Delta _{\Omega _1}^N & 0 & 0 \\ 0 & 0 & D_{u_2} \Delta _{\Omega _2}^N & 0 \\ 0 & 0 & 0 & D_{v_2} \Delta _{\Omega _2}^N \end{pmatrix}, \quad \mathcal {D}(A) = \prod _{i=1}^2 \prod _{k=1}^2 \mathcal {D}(\Delta _{\Omega _i}^N), $$

where $\Delta _{\Omega _i}^N$ is the Laplacian on $\Omega _i$ with Neumann boundary conditions. The operator *A* generates a $C_0$-semigroup on $X = \prod _{i=1}^2 \mathcal {C}^0(\overline{\Omega _i})^2$, allowing the coupled system to be written in abstract form

$$ \partial _t U = A U + G(U), \quad U(0) = U_0 \in X^+, $$

a framework in which Proposition 2.10 guarantees the existence of a unique maximal solution.

Our prey-predator model on network described by (15) additionally satisfies the following property:

##### Proposition 3.1

For all $U_0 \in X^+$, the solution $U=(u_1,v_1,u_2,v_2)$ of problem (15) is globally bounded.

##### Proof

The proof relies on viewing each subsystem of the network (15) as a perturbed single-domain predator-prey system. Consider the following system on a bounded domain Ω with smooth boundary:

16 $$\begin{aligned} {\left\{ \begin{array}{ll} \displaystyle \partial _t u = D_u \Delta u + u(1-u) - \alpha u v - m u p(x) + g_1(t,x), & t>0, \ x \in \Omega ,\\ \displaystyle \partial _t v = D_v \Delta v + \alpha u v - \mu v - m v p(x) + g_2(t,x), & t>0, \ x \in \Omega ,\\ \partial _\eta u = \partial _\eta v = 0, & t>0, \ x \in \partial \Omega ,\\ u(0,x) = u_0(x), \quad v(0,x) = v_0(x), & x \in \Omega , \end{array}\right. } \end{aligned}$$

where *p*, $g_1$, and $g_2$ are positive and bounded functions, and $(u_0,v_0)$ are positive initial conditions.

Each subsystem of (15) can be written in the form (16), with the coupling terms replaced by the perturbation functions *p*, $g_1$, and $g_2$.

The main difficulty lies in proving that a system of type (16) admits a solution that remains positive and globally bounded. Although the solution is formulated in the mild sense, it is assumed here to be a classical solution. Indeed this can be established by classical results on boundedness of reaction-diffusion systems, see Morgan (1990). Positivity follows from the quasi-positivity of the reaction terms, while boundedness is ensured by the dissipative structure of the logistic and predator-prey interactions.

Since the number of subsystems is finite (n=2) and each solution remains positive and bounded, it immediately follows that the solution $U=(u_1,v_1,u_2,v_2)$ of the full coupled system (15) also remains positive and globally bounded. □

In the following we present some numerical results we obtained for two and three node networks. For the discretization of our nonlocal model, we employed the finite element method, utilizing a splitting technique to efficiently manage the different operators (Descombes 2001). Simulations have been performed using Freefem++ software (see Hecht et al. (2005)). The simulation outputs were stored in formats compatible with ParaView and R, facilitating detailed visualization and analysis of the results. ,

#### Numerical simulations for the two habitats prey-predator models

**Fig. 2:**
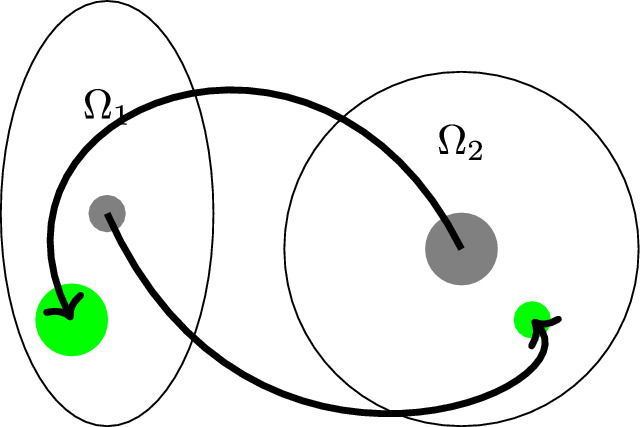
Spatial interaction between two habitats with departure zones shown in grey and arrival zones in green.

In this subsection, we will simulate the interaction of two prey-predator systems, each living in a 2-dimentional domain $\Omega _i$ (i=1,2). As it can be seen in Figure 2, the first domain, which we will denote as $\Omega _1$, has an elliptical shape, while the second domain, noted as $\Omega _2$, has a circular form. Within $\Omega _1$, we will examine the evolution of prey $u_1$ and predator $v_1$ populations, and similarly $u_2$, $v_2$ in $\Omega _2$.

**Fig. 3:**
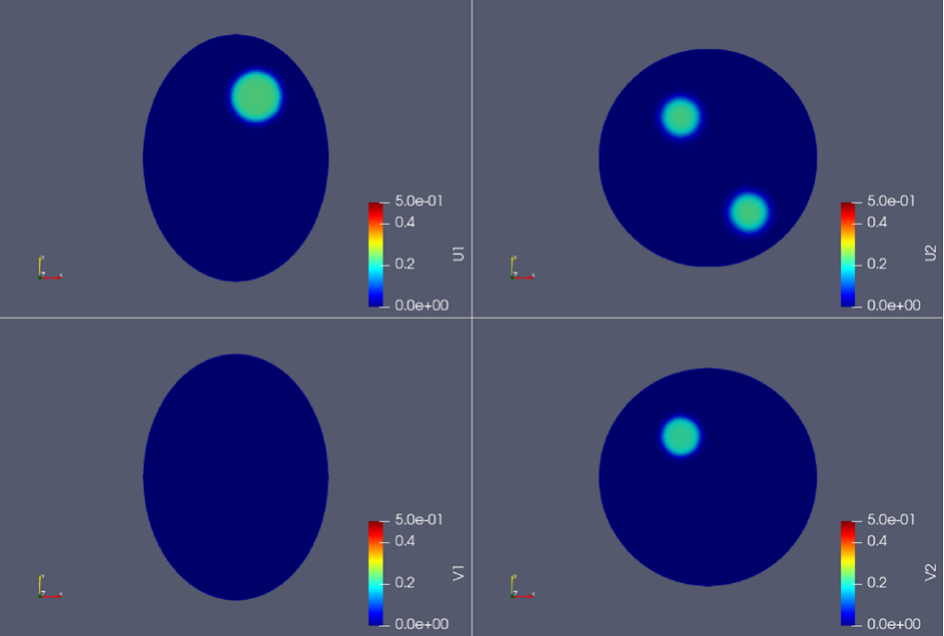
Representation of initial conditions: areas where population density is highly concentrated are shown in green, while those where it is less concentrated or even negligible are shown in blue. At the top left, we have the prey population in the domain $\Omega _1$. At the bottom left, the predator population (initially zero). At the top right, we have the prey divided into two zones $\in \Omega _2$, and at the bottom right, the initial predator density.

For each domain $\Omega _1$ and $\Omega _2$, we define a starting zone, corresponding to a part of the domain where $P_{ji} \ne 0$ for i≠j, with $i, j \in \{1,2\}$, (we recall that $P_{ji}(x_i)$ is the probability of moving from $x_i$ to $\Omega _j$). Additionally, each domain $\Omega _i$ includes an arrival zone where $\varepsilon _i \ne 0$, for $i \in \{1,2\}$ (see Figure  2). For $i, \ j \in \{1, 2\}$, i≠j, we use indicator functions to define the probabilities $P_{ji}$ and $\varepsilon _i$.

For the numerical simulations, we choose the following scenario. At the initial time, we assume that on the domain $\Omega _1$, there are no predators, only prey grouped in one location (see Figure 3). On $\Omega _2$, we choose to have a prey population twice as dense as that of predators, and we distribute the prey population into two zones within the domain $\Omega _2$, while the predator population is concentrated in one location in $\Omega _2$ (see Figure 3). We promote migration from domain $\Omega _2$ to $\Omega _1$ by allowing a maximum quantity of individuals migrating from $\Omega _2$ to $\Omega _1$ that is twice as large as the migration from $\Omega _1$ to $\Omega _2$. Although the model coefficients may differ at each node, we consider a scenario where the model coefficients are equal in pairs to better understand the effects of coupling (see Table  1).

**Table 1:** Summary table of the parameters chosen for the simulations.

Parameters	Node 1	Node 2
Coupling coefficient	$\alpha _{21}=0.45$	$\alpha _{12}=0.9$
($D_u$, $D_v$)	(0.2, 0.1)	(0.2, 0.1)
(α, μ)	(0.3, 0.1)	(0.3, 0.1)

**Fig. 4:**
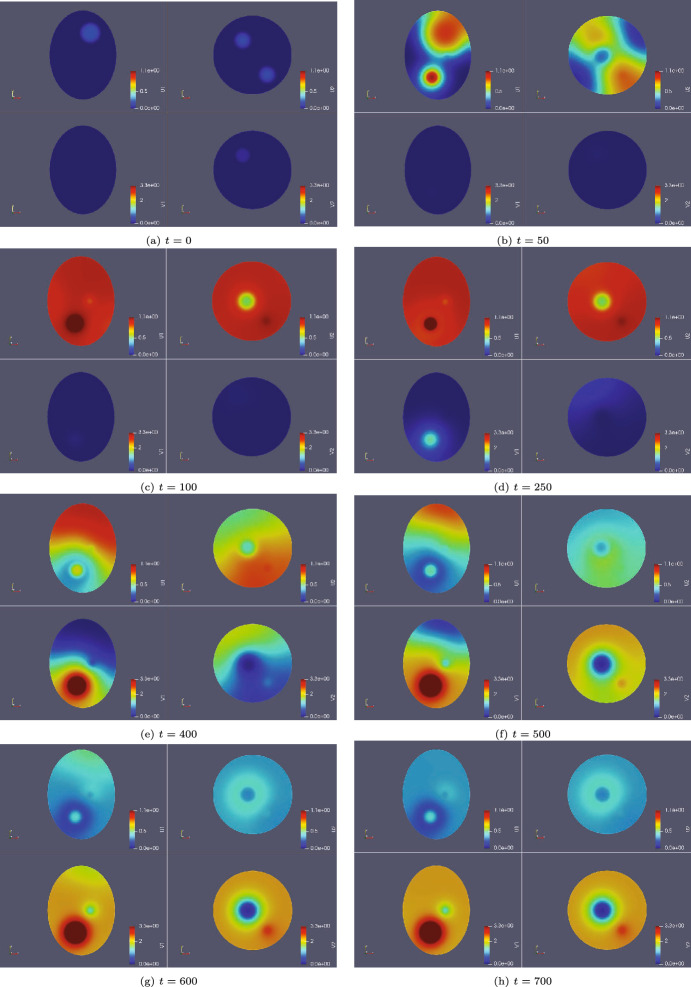
Evolution of the densities of different populations over time: the higher the population density, the more the colors range from blue to red. (The visualization of prey and predator populations does not occur on the same scale). Starting (t=0) from a fairly low initial condition for $u_1$ in a single location, zero for $v_1$, fairly low for $u_2$ but in two locations, and very low for $v_2$ in a single location, we can see that the densities of $u_1$ and $u_2$ rise sharply to become very high (t=100), then asymptotically, around t=600, they converge to non-homogenous state, and stabilize at medium densities for $u_1$ and $u_2$ and fairly high densities for $v_1$ and $v_2$. This is also confirmed in figure  5 below.

**Fig. 5:**
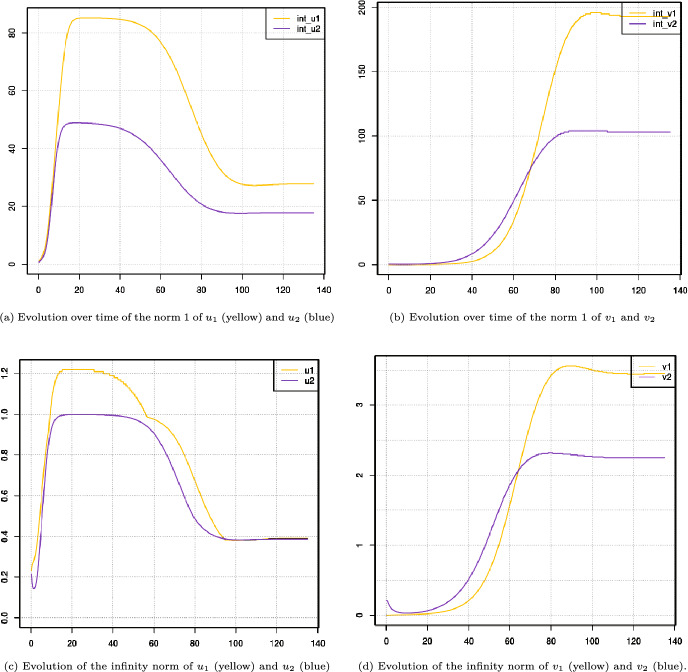
Evolutions over time of the norm 1 and infinity norm of the densities of the prey and predator.

In Figure 4, the higher the population density, the more the colors range from blue to red. The simulation results convincingly highlight the phenomena modeled in this study, namely diffusion, predation, and migration. Indeed, in Fig  4, at t=50, the impact of migration on population $u_1$ is clearly noticeable, as well as the effect of diffusion on populations $u_1$ and $u_2$. At t=250, migration continues to affect populations $u_1$, $u_2$, and $v_1$, and the departure and arrival zones are clearly observable. By t=500, in addition to the clearly visible phenomena of diffusion and migration, the emergence of the predation phenomenon is notable, manifested by the decrease in prey density where a high density of predators is observed. Furthermore, between t=600 and t=700, it is observed that the model solution converges to a non-homogeneous equilibrium state. Figure  5 shows that the solution of the coupled system remains bounded in both the norm 1 and norm ∞.

### The case of a three habitats network model

**Fig. 6:**
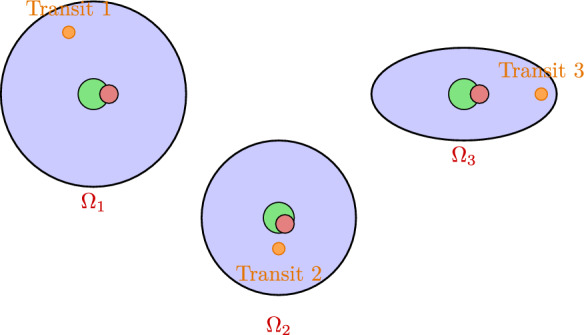
Schematic representation of the three-node network of the three domains $\Omega _1$, $\Omega _2$, and $\Omega _3$. The locations of the initial populations $u_i$ (depicted in green) and $v_i$ (depicted in red), along with the transit zones (shown in orange), are illustrated herein. The circles and the ellipse illustrate the geometrical structure of the domains.

We consider now a network composed of three interconnected spatial domains, denoted by $\Omega _1$, $\Omega _2$, and $\Omega _3$. On each domain evolve two interacting populations: a prey population $u_i$ and a predator population $v_i$ ($i\in \{1, 2, 3\}$). The geometrical configuration of the network is defined as follows: $\Omega _1$ is a disk of radius $r_1 = 3$; $\Omega _2$ is a disk of radius $r_2 = 2.5$; and $\Omega _3$ is an ellipse centered with semi-major and semi-minor axes $a_3 = 3.0$ and $b_3 = 1.5$, respectively (see Figure 6). Each domain contains a specific transit zone where departure and arrival of individuals are allowed. This time, we assume that the departure and arrival zones coincide, such that the arrival probability on $\Omega _i$, denoted by $\varepsilon _i(x_i)$, and the departure probability from $\Omega _i$ to $\Omega _j$, denoted by $p_{ji}(x_i)$, are given by

$$ \varepsilon _i(x_i) = p_{ji}(x_i) = \exp \!\left( -\frac{\Vert x_i - x_i^*\Vert ^2}{\rho _i^2}\right) , $$

for $x_i \in \Omega _i$ and $i,j \in \{1,2,3\}$, i≠j, where

$$ x_1^* = (-0.8, 2), \quad x_2^* = (0, -1), \quad x_3^* = (2.5, 0), \quad \text {and} \quad \rho _i = 0.2. $$

The local dynamics within each domain are governed by a Lotka-Volterra-type reaction-diffusion system with nonlocal coupling:

$$ {\left\{ \begin{array}{ll} \partial _t u_i = D_{u_i} \Delta u_i + u_i(1 - u_i) - \alpha _i u_i v_i + \displaystyle \sum _{j \in I^{in}_i} \alpha _{u,ij}\, \varepsilon _i(x_i) \\ \quad \displaystyle \int _{\Omega _j} p_{ij}(x_j)\, u_j(x_j)\, dx_j - \displaystyle \sum _{j \in I^{out}_i}\alpha _{u,ji}\, p_{ji}(x_i)\, u_i(x_i), \\ \partial _t v_i = D_{v_i} \Delta v_i + \alpha _i u_i v_i - \mu _i v_i +\displaystyle \sum _{j \in I^{in}_i}\alpha _{v,ij} \,\varepsilon _i(x_i) \\ \quad \displaystyle \int _{\Omega _j} p_{ij}(x_j)\, v_j(x_j)\, dx_j - \displaystyle \sum _{j \in I^{out}_i} \alpha _{v,ji} \,p_{ji}(x_i)\, v_i(x_i), \end{array}\right. } $$

where $D_{u_i}$ and $D_{v_i}$ denote the diffusion coefficients, and $\alpha _{u,ji}$, $\alpha _{v,ji}$ represent the migration coefficients of preys and predators from $\Omega _i$ to $\Omega _j$, respectively. The parameters $\alpha _i$ (predation rate) and $\mu _i$ (predator mortality rate) can be chosen so that, in the absence of coupling, the isolated system admits a stable coexistence equilibrium $(u_i^*, v_i^*)$ with both species present (if $\alpha _i > \mu _i$), or exhibits an equilibrium with predator extinction $(u_i^*, v_i^* = 0)$ (for instance, if $\alpha _i < \mu _i$).

The initial distributions of $u_i$ and $v_i$ are specified as localized patches within each domain. When present, the prey populations $u_i$ are initialized inside a disk of radius 0.5 with a total mass of 0.1, while the predator populations $v_i$ are distributed inside a disk of radius 0.3 with a total mass of 0.2. These disks represent the initial spatial distributions of the populations, and their sizes reflect the magnitude of the initial masses. Figure 6 displays the geometric configuration of the network and the initial population locations.

### Scenario 1: Asymmetric network with coexistence and partial extinction

**Fig. 7:**
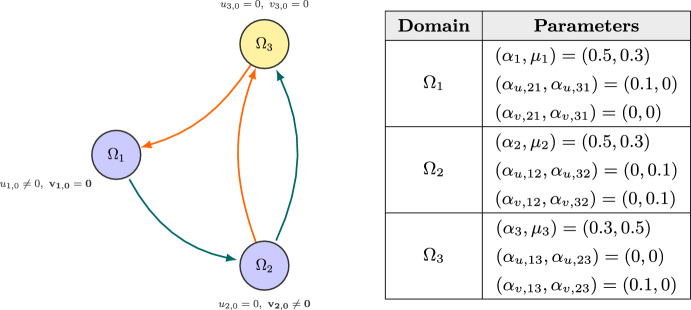
Scenario 1-Predator invasion through asymmetric coupling. Configuration of the three-domain network $\Omega _i$ illustrating the fluxes of prey *u* (blue) and predators *v* (orange). Arrows indicate the direction of exchanges between domains; a missing arrow implies a zero coupling coefficient in that direction. Local parameters are chosen so that, in isolation, the domains $\Omega _1$ and $\Omega _2$ admit a stable coexistence equilibrium $(u_i^*, v_i^*)$ of prey and predators with $(\alpha _i, \mu _i) = (0.5, 0.3)$, while domain $\Omega _3$, characterized by $(\alpha _3,\mu _3)=(0.3,0.5)$, tends to predator extinction when isolated, providing an interesting test case for cross-domain dynamics.

**Fig. 8:**
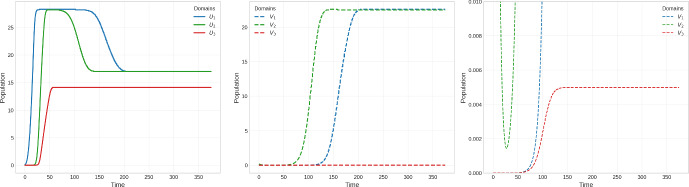
Scenario 1-Time evolution (norm 1) of the prey populations $u_i$ (left), the predator populations $v_i$ (center), and a zoom on the predators (right) for the three domains $\Omega _i$ ($i\in \{1,2,3\}$).

**Fig. 9:**
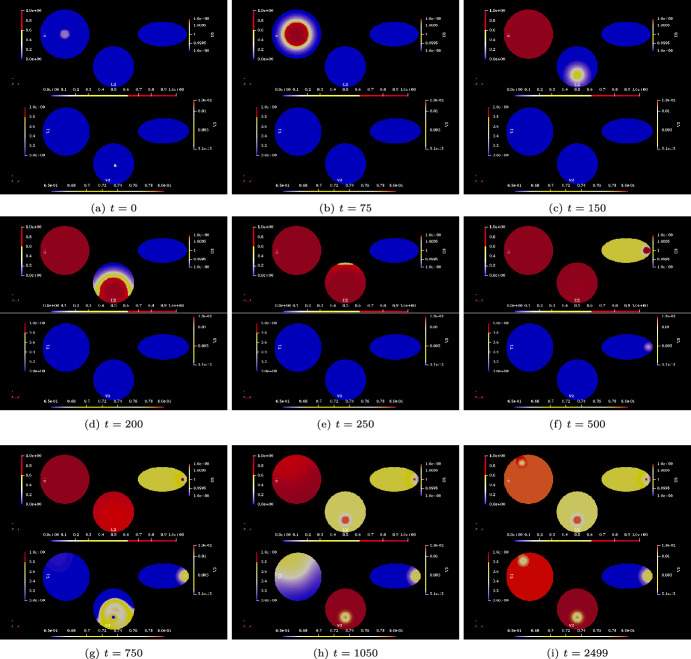
Scenario 1-Spatial evolution of prey and predator populations at selected time instants. Each panel displays the density distribution of prey (top) and predator (bottom) populations at the considered time *t*. The colorbar associated with each spatial distribution indicates the observation scale of the densities. These scales may vary, even for the same population, in order to highlight phenomena occurring at different spatial scales at the same instant over distinct domains.

This first scenario serves as a baseline configuration for the subsequent experiments. Fig 7 It is designed to highlight how nonlocal, directed migrations can generate coexistence and recolonization phenomena at the network scale, despite strongly unbalanced initial conditions and locally unfavorable dynamics on some nodes. Initially, prey populations are present only in $\Omega _1$, predator populations only in $\Omega _2$, while $\Omega _3$ is empty. Local parameters are chosen so that, in isolation, domain $\Omega _3$ cannot sustain predators and converges toward predator extinction (see Fig. 7). Despite these highly asymmetric initial conditions, the numerical simulations in Fig. 8 reveal an emergent collective behavior induced by the network structure and the nonlocal migration fluxes. The system converges toward a regime of long-term coexistence across the entire network. The spatial snapshots in Fig. 9 show that populations tend to concentrate around the transit zones, highlighting the central role played by nonlocal exchanges. The colorbar displayed next to each spatial distribution indicates the density scale used for visualization. Different scales are employed across domains for the same population in order to highlight phenomena occurring at different intensity levels at the same time. These simulations clearly illustrate a network-induced ecological rescue effect: the coupling mechanism allows the persistence of viable populations in a habitat ($\Omega _3$) that would be inviable in isolation. In this configuration, $\Omega _3$ acts as a dynamic sink, which not only receives individuals but also serves as an indirect redistribution hub through which predator populations can be relayed toward other domains. As a consequence, the system converges toward spatially non-homogeneous equilibrium states, resulting from the localization of nonlocal exchanges around the transit zones. These equilibria persist despite diffusion and are similar to those observed in the two-node network case, revealing a coupling-induced symmetry-breaking mechanism.

### Scenario 2: Removal of the predator flux from $\Omega _3$ to $\Omega _1$

**Fig. 10:**
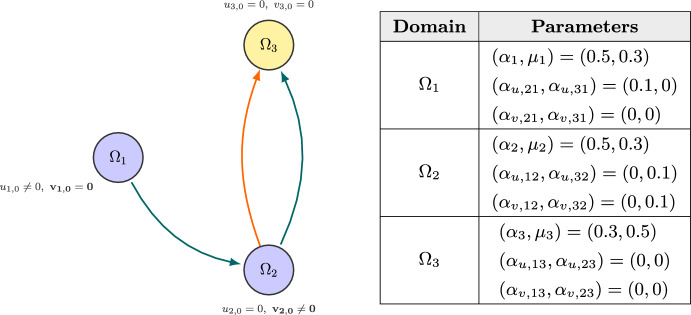
Scenario 2-Failure of predator invasion under asymmetric coupling. Configuration of the three-domain network $\Omega _i$ illustrating the fluxes of prey *u* (blue) and predators *v* (orange). Arrows indicate the direction of exchanges between domains; a missing arrow implies a zero coupling coefficient in that direction. Local parameters are chosen so that, in isolation, the domains $\Omega _1$ and $\Omega _2$ admit a stable coexistence equilibrium $(u_i^*, v_i^*)$ of prey and predators with $(\alpha _i, \mu _i) = (0.5, 0.3)$, while domain $\Omega _3$, characterized by $(\alpha _3,\mu _3)=(0.3,0.5)$, tends to predator extinction when isolated, providing an interesting test case for cross-domain dynamics.

**Fig. 11:**
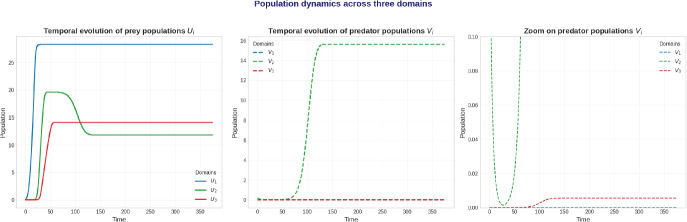
Scenario 2-Time evolution (norm 1) of the prey populations $u_i$ (left), the predator populations $v_i$ (center), and a zoom on the predators (right) for the three domains $\Omega _i$ ($i\in \{1,2,3\}$).

**Fig. 12:**
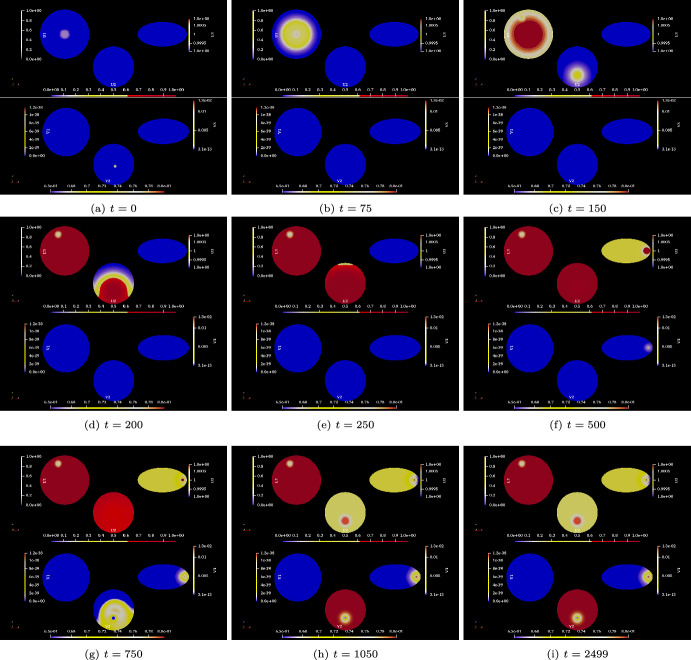
Scenario 2-Spatial evolution of the prey and predator populations at selected time steps. Each panel shows the density distribution of prey (top) and predator (bottom) populations at the corresponding instant *t*. The colorbar associated with each spatial distribution indicates the observation scale of the densities. These scales may vary, even for the same population, in order to highlight phenomena occurring at different spatial scales at the same instant over distinct domains.

In this second scenario, we keep exactly the same geometric configuration, local parameters, Fig 10 and initial conditions as in Scenario 1. The only difference lies in the modification of the nonlocal migration structure: the predator flux from $\Omega _3$ to $\Omega _1$ is suppressed, while all other migration fluxes are kept unchanged (see Fig. 10). This scenario is designed to assess the role of this single directed flux in the global network dynamics. The temporal evolutions in Fig. 11 show that prey populations exhibit dynamics that are globally similar to those observed in Scenario 1. Predator dynamics, however, are strongly affected: no predator colonization of $\Omega _1$ is observed, and the synchronization previously observed between $v_1$ and $v_2$ in Scenario 1 is lost. From a spatial perspective (see Fig. 12) predators remain strictly confined to their source domains, and no predator density is observed in $\Omega _1$ at any time. Although $\Omega _3$ still acts as a sink, it loses its role as an indirect relay node, which prevents any effective redistribution of predators toward the rest of the network. This scenario highlights the decisive role played by the flux $\Omega _3 \rightarrow \Omega _1$ in indirect predator diffusion and in the emergence of coupled dynamics at the network scale.

#### Comparison between Scenarios 1 and 2: Role of migration topology

Scenarios 1 and 2 share identical geometric configurations, local parameters, and initial conditions. The only difference between them is the suppression, in Scenario 2, of the predator migration flux from $\Omega _3$ to $\Omega _1$. This targeted modification allows us to isolate the role of this directed coupling in the global network dynamics. In Scenario 1, the network exhibits a robust long-term coexistence of prey and predator populations across all nodes, despite highly asymmetric initial conditions and locally unfavorable dynamics in $\Omega _3$. The presence of directed nonlocal migrations enables an effective redistribution of individuals at the network scale, leading to the emergence of non-homogeneous equilibrium states. In particular, predator populations synchronize between $\Omega _1$ and $\Omega _2$, revealing a strong collective behavior induced by the coupling structure. In contrast, Scenario 2 shows that removing the predator flux $\Omega _3 \rightarrow \Omega _1$ profoundly alters the predator dynamics, while leaving prey dynamics largely unchanged. Predators fail to colonize $\Omega _1$, and the synchronization previously observed between $v_1$ and $v_2$ disappears. Spatially, predator densities remain confined to their source regions, and $\Omega _3$ loses its role as an indirect redistribution node. These results demonstrate that species coexistence at the network scale is not solely determined by local dynamics or by the presence of migration, but critically depends on the directionality and completeness of the migration topology. In particular, the flux $\Omega _3 \rightarrow \Omega _1$ plays a decisive role in enabling indirect diffusion mechanisms, synchronization effects, and the persistence of predators across the entire network.

### Scenario 3: Effect of heterogeneous transit zone sizes

Scenario 3 preserves the migration topology of Scenario 1 but introduces a marked spatial heterogeneity through modifications of the transit zone radii. This geometric variation directly impacts the effective intensity of nonlocal exchanges. Unlike scenarios 1, the radii of the transit zones are no longer uniform and are chosen as follows:

- on node $\Omega _1$, the transit zone radius is increased from 0.2 to 0.75,
- on node $\Omega _2$, the transit zone radius is increased from 0.2 to 1.4,
- on node $\Omega _3$, the transit zone radius is decreased from 0.2 to 0.1.

**Fig. 13:**
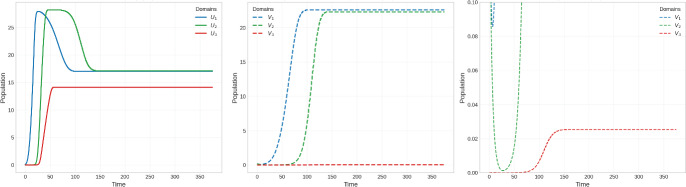
Scenario 3 - Time evolution (norm 1) of the prey populations $u_i$ (left), the predator populations $v_i$ (center), and a zoom on the predators (right) for the three domains $\Omega _i$ ($i\in \{1,2,3\}$).

**Fig. 14:**
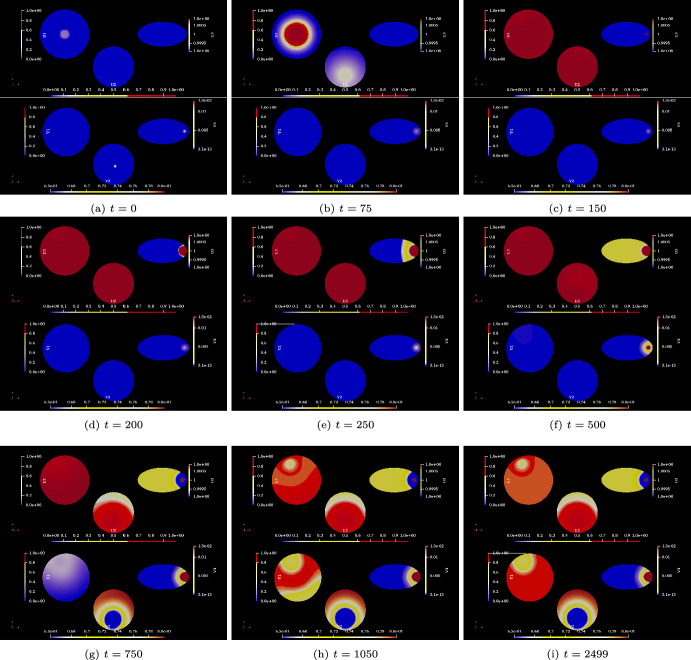
Spatio-temporal evolution of the prey and predator populations at selected time steps. Each panel shows the density distribution of prey (top) and predator (bottom) both populations at the corresponding instant *t*. The colorbar associated with each spatial distribution indicates the observation scale of the densities. These scales may vary, even for the same population, in order to highlight phenomena occurring at different spatial scales at the same instant over distinct domains.

The temporal evolutions in Fig. 13 show that the significant enlargement of the transit zones in $\Omega _1$ and $\Omega _2$ strengthens the associated migration fluxes, leading to a faster increase and higher population levels in these domains. Conversely, the reduction of the transit zone in $\Omega _3$ limits its ability to receive and redistribute individuals, rendering its dynamics more marginal at the network scale. The spatio-temporal evolutions in Fig. 14 reveal an increased concentration of densities around the enlarged transit zones, confirming that the geometry of the exchange regions constitutes a major control parameter of the global dynamics. This geometric effect directly translates into spatially heterogeneous equilibrium states at the network scale, even though the migration topology and local dynamics remain unchanged.

#### Comparison between Scenarios 1 and 3: Effect of transit zone geometry

Scenarios 1 and 3 share the same migration topology, local parameters, and initial conditions. The only difference lies in the geometry of the transit zones, which are uniform in Scenario 1 and heterogeneous in Scenario 3. This comparison allows us to isolate the impact of spatial geometry on nonlocal migration processes and on the resulting network dynamics. In Scenario 1, uniform transit zones lead to balanced nonlocal exchanges between domains, promoting a coherent redistribution of populations across the network. The resulting equilibrium states are non-homogeneous but remain dynamically coupled, with all nodes contributing to long-term species coexistence. In Scenario 3, enlarging the transit zones in $\Omega _1$ and $\Omega _2$ significantly strengthens the associated migration fluxes, accelerating population growth and leading to higher equilibrium densities in these domains. Conversely, the reduced transit zone in $\Omega _3$ weakens its connectivity, making its dynamics more marginal and less influential at the network scale. The spatial distributions reveal a pronounced concentration of densities around the enlarged transit zones, resulting in more heterogeneous equilibrium states. These results highlight that the geometry of the exchange regions constitutes a key control parameter of the global dynamics, capable of modulating both the intensity of nonlocal interactions and the spatial structure of equilibrium states, even when migration topology and local dynamics are unchanged.

#### General synthesis of the three scenarios

The numerical simulations presented in this work illustrate the richness of dynamical behaviors that can emerge in networks of reaction–diffusion systems coupled through nonlocal, directed migration processes. Across the three scenarios considered, the results consistently demonstrate that long-term species coexistence is a genuinely network-induced phenomenon, which cannot be predicted solely from local dynamics or isolated domain behavior. Scenario 1 shows that directed nonlocal migrations can overcome strongly asymmetric initial conditions and locally unfavorable dynamics, leading to network-wide coexistence and to the emergence of non-homogeneous equilibrium states. The coupling structure induces collective behaviors such as indirect diffusion and synchronization, and gives rise to a clear network-induced ecological rescue effect. Scenario 2 highlights the critical importance of migration topology and directionality. The removal of a single directed flux is sufficient to suppress predator colonization in one domain, destroy synchronization effects, and significantly weaken coexistence at the network scale. This emphasizes that partial connectivity may be insufficient to sustain global persistence. Scenario 3 demonstrates that, beyond topology, the geometry of the exchange regions plays a fundamental role in shaping the global dynamics. Heterogeneous transit zones lead to uneven redistribution of populations and reinforce spatial heterogeneity in equilibrium states, confirming that geometric features act as effective control parameters of nonlocal interactions. Overall, these simulations confirm that the interplay between migration topology, directionality, and spatial geometry governs both the persistence of species and the structure of equilibrium states in PDE networks. They provide strong numerical evidence supporting the theoretical framework developed in this work and open perspectives for the modeling of complex ecological, biological, and socio-dynamical systems with spatially structured interactions.

## Conclusion

In this work we have proposed a new mathematical framework to model the spatio-temporal dynamics of several populations interacting each others on disjoint domains. The migration processes are captured via non-local coupling terms. The non-local approach stands out for its unique ability to model long-distance interactions between spatially disjoint domains, offering increased flexibility in the shape, size and dimension of the domains.

First of all, we have presented the model equations in detail, in both the scalar and the vectorial cases. Then, a theoretical analysis of these models have been developed, by showing that that several properties of the network model can be derived from the ones of the model on the single node.

As case test, the exemple of a prey-predator model on two and three disjoint domains has been theoretically and numerically investigated. In particular, the simulation results effectively capture the different dynamics we aim to model, namely diffusion, predation and migration. Furthermore, we have shown that the network’s connectivity properties and nonlocal coupling influence species survival, even in areas that would otherwise be unsuitable for certain species. These simulations allow us to illustrate the proposed partial differential equations coupling method and clearly demonstrate its faithful modeling of interactions between different domains. However, given the existence of non-homogeneous equilibrium states, a theoretical study on determining equilibrium states and studying their stability is more than necessary to confirm the observed results.

## Supplementary Information

Below is the link to the electronic supplementary material.

Supplementary file 1 (edp 22 KB)

## Appendix A. Abstract formulation of (10)

The aim of the Appendix is to reformulate Problem (10) as an Abstract Cauchy Problem and prove Proposition 2.10. Note that Proposition () will be obtained as a special case of this analysis since System (7) is contained in (10) with m=1.

To consider (10), let first introduce, for each i=1,..,n the space $\mathcal {C}\left( \overline{\Omega }_i\right)$ endowed with the usual sup-norm. We also denote by $\mathcal {C}\left( \overline{\Omega }_i; \mathbb {R}^+\right)$ its positive cone which consists in the set of continuous and nonnegative functions.

Recall that from Corollary 3.1.24 (ii) in Lunardi (2012), the following results holds.

### Lemma A.1

For i=1,..,n, for any d>0 and any for $m\in \mathcal {C}^1\left( \overline{\Omega }_i\right)$ with m(x)≥0 for all $x\in \partial \Omega$, the linear operator $A=A(d,m,\Omega _i):D(A)\subset \mathcal {C}\left( \overline{\Omega }_i\right) \rightarrow \mathcal {C}\left( \overline{\Omega }_i\right)$ given by

$$\begin{aligned} D(A)=\left\{ u\in \bigcap _{p\ge 1}W^{2,p}\left( \Omega _i\right) :\;u,\;\Delta u\in \mathcal {C}\left( \overline{\Omega }_i\right) ,\;\partial _{\eta _i}u(x_i)=-m(x_i)u(x_i)\;\,x_i\in \partial \Omega _i\right\} , \end{aligned}$$

with $Au(x_i)=d\Delta u(x_i),\;x_i\in \overline{\Omega }_i$ is densely defined and generates a strongly continuous, analytic and positive semigroup. Here by positive semigroup, we means that it maps $\mathcal {C}\left( \overline{\Omega }_i; \mathbb {R}^+\right)$ into itself.

Using the above lemma, we define for each i=1,..,n and k=1,..,m the linear operator $(A_i^k, D(A_i^k))$ on $\mathcal {C}\left( \overline{\Omega }_i\right)$ by

$$ A_i^k=A\left( D_i^k, \tilde{m}_i^k,\Omega _i\right) , $$

and we denote by $\left\{ T_{A_i^k}(t)\right\} _{g\ge 0}$ the positive semigroup generated by $A_i^k$ on $\mathcal {C}\left( \overline{\Omega }_i\right)$.

Now to go further, for any i=1,..,n, we define the Banach space $X_i$ by

$$ X_i=\left( \mathcal {C}\left( \overline{\Omega }_i\right) \right) ^m, $$

endowed with the usual product norm, its positive cone $X_{i,+}:=\left( \mathcal {C}\left( \overline{\Omega }_i; \mathbb {R}^+\right) \right) ^m$ as well as the linear operator $A_i: D\left( A_i\right) \subset X_i\rightarrow X_i$ by

$$ D(A_i)=\prod _{k=1}^m D(A_i^k)\text { and }A_i=\textrm{diag}\;\left( A_i^1,\cdots ,A_i^m\right) . $$

Here and below $\textrm{diag}$ means the diagonal matrix. Due to the above result about the generation of semigroup, note that $A_i$ is also the infinitesimal generator of strongly continuous, analytic and positive semigroup, denoted by $\left\{ T_ {A_i}(t)\right\} \subset \mathcal {L}(X_i)$ and given by

$$ T_{A_i}(t)=\textrm{diag}\;\left( T_{A_i^1}(t),\cdots ,T_{A_i^m}(t)\right) ,\;\;\forall t\ge 0. $$

Next, we introduce the Banach space $X:=X_1\times \cdots \times X_n$, endowed with the product norm, which will allow us to coupled the dynamics of the different nodes, $\Omega _1$, ..., $\Omega _n$. We also introuce its positive cone $X_+$ given by $X_+=X_{1,+}\times \cdots \times X_{n,+}$. A generic point U∈X is written as the vector $\left( U_1,\cdots , U_n\right) ^T$ with $U_i\in X_i$ while $U_i$ is written component by component as $U_i=\left( U_i^1,\cdots , U_i^m\right) ^T$. Next to rewrite (10) as an Abstract Cauchy Problem on *X*, we consider the nonlinear map G:X→X given by

$$\begin{aligned} G(U)=\left( G_1(U),\cdots , G_n(U)\right) ^T\text { with } G_i:X\rightarrow X_i \end{aligned}$$

with, for $$U=(U_i^k)\in X$$,

$$\begin{aligned} {\begin{matrix} G_i(U)(x_i)=& \begin{pmatrix} F_i^1\left( U_i^1(x_i),\cdots ,U_i^m(x_i)\right) \\ \ldots \\ F_i^m\left( U_i^1(x_i),\cdots ,U_i^m(x_i)\right) \end{pmatrix} -\begin{pmatrix}m_i^1(x_i) U_i^1(x_i)\\ \ldots \\ m_i^m(x_i) U_i^m(x_i)\end{pmatrix}+\begin{pmatrix} \theta _i^1(U,x_i)\\ \ldots \\ \theta _i^m(U,x_i)\end{pmatrix}. \end{matrix}} \end{aligned}$$

Here recall that $\theta _i^k:X\rightarrow \mathcal {C}\left( \overline{\Omega }_i\right)$ is given by

$$ \theta _i^k(U)(x_i) =\varepsilon _i^k(x_i)\left[ \sum \limits _{{j \in I_i^{in}}} \int _{\Omega _j}m_{ij}^k(x_j)U_j^k(x_j)dx_j+\sum \limits _{{j \in B_i^{in}}} \int _{\partial \Omega _j}\tilde{m}_{ij}^k(x_j)U_j^k(x_j)dx_j\right] . $$

Equipped together with the above notations, Problem (10) rewrites as the following Abstract Cauchy Problem on *X*:

A.1 $$\begin{aligned} \frac{dU(t)}{dt}=AU(t)+G(U(t)),\;t>0\text { and }U(0)=\left( U_{i,0}^k\right) _{\begin{array}{c} i=1,..,n\\ k=1,..,m \end{array}}\in X. \end{aligned}$$

We assume the following property for the function $F=(F_1,\cdots , F_n)$:

(HF): For all i=1,..,n, function $F_i:\mathbb {R}^m\rightarrow \mathbb {R}$ is of class $\mathcal {C}^1$ and satisfies for each M>0, there exists λ(M)>0 such that for all $u=(u_1,..,u_m)\in \mathbb {R}^m$ with $u_1\ge 0$,...,$u_m\ge 0$ with $u_1+\cdots u_m\le M$ we have $F_i(u_1,\cdots ,u_m)+\lambda _i(M)u_i\ge 0.$

Under the above assumption we readily obtain the following result.

### Proposition A.2

Under the above assumption, for all initial data $U_0\in X_+$, there exists a maximal existence time $T_\textrm{max}>0$ and a unique function $U\in \mathcal {C}\left( \left[ 0,T_\textrm{max}\right) ; X_+\right)$ such that

A.2 $$\begin{aligned} U(t)=T_{A}(t)U_0+\int _0^t T_{A}(t-s)G(U(s))ds,\,\,\forall t\in \left[ 0,T_\textrm{max}\right) . \end{aligned}$$

Moreover, either $T_\textrm{max}=\infty$, either $T_\textrm{max}<\infty$ and

$$ \lim _{t\nearrow T_\textrm{max}} \Vert U(t)\Vert _X=\infty . $$

To check the above result, one can first notice that that the map *G* is locally Lipschitz continuous on *X* so that Theorem 1.4 of Pazy (1983) applies and ensures the existence of a maximum time interval and of a unique weak solution of (A.1), in the sense of (A.2). The positivity of the solutions can be obtained by applying results in Chapter 5.1 in the book of Magal and Ruan (2018).

### Proof

The proof follows standard semigroup theory combined with positivity arguments.

Step 1: Local existence via semigroup theory. Let *A* denote the linear operator generating the strongly continuous and positive semigroup $T_A(t)$ on *X*, and let G:X→X be the nonlinear map encoding the local interactions and nonlocal migration. Since *G* is locally Lipschitz on *X*, chapter 5 theorem 1.4 in Pazy (1983) ensures the existence of a maximal solution *U* of (A.1) on a time interval $[0,T_\textrm{max})$ and uniqueness in the sense of (A.2).

Step 2: Positivity of the solution. To ensure $U(t) \in X_+$ for all $t \in [0,T_\textrm{max})$, we verify Assumption 5.3.1 of Magal and Ruan (2018), as suggested by Proposition 5.3.2 therein.

i) Consider a bounded subset of $X_+$, $\overline{B_{X^+}(0,M)} = \{ u \in X_+: \Vert u\Vert _X \le M \}$.
ii) For $u \in \overline{B_{X^+}(0,M)}$, there exists $\lambda _M > 0$ such that
  $$\begin{aligned} G(u) + \lambda _{M} u \ge 0. \end{aligned}$$
  This is obtained by bounding the local interaction term $F_i(u_i)$ and using the non-negativity of the migration coefficients $m_{ij}, \tilde{m}_{ij}$ and the weights $\varepsilon _i$.
iii) Define the bounded linear operator $B = \textrm{diag}(\lambda _M^1, \dots , \lambda _M^n)$. Then A-γB generates a positive semigroup and $G(u) + B u \in X_+$ for all $u \in X_0^+:= \overline{\mathcal {D}(A)} \cap X_+$.

By Proposition 5.3.2 of Magal and Ruan (2018), this ensures that the solution *U*(*t*) remains in $X_+$ for all $t \in [0,T_\textrm{max})$.

Step 3: Conclusion. Combining the above steps, we conclude that for any initial data $U_0 \in X_+$, there exists a unique, positive solution of (A.1) defined on $[0,T_\textrm{max})$. □
